# Association between falls in elderly and the number of chronic diseases and health-related behaviors based on CHARLS 2018: health status as a mediating variable

**DOI:** 10.1186/s12877-022-03055-x

**Published:** 2022-04-28

**Authors:** Shaoliang Tang, Meixian Liu, Tongling Yang, Chaoyu Ye, Ying Gong, Ling Yao, Yun Xu, Yamei Bai

**Affiliations:** 1 School of Health Economics and Management, Nanjing University of Chinese Medicine, Nanjing, China; 2 School of Nursing, Nanjing University of Chinese Medicine, Nanjing, China

**Keywords:** Chronic diseases, Health-related behaviors, Fall, Health status, Mediation effect

## Abstract

**Objective:**

Falling is one of the main causes of death and morbidity in the elderly. This study aims to explore the association between elderly patients with chronic diseases and their health-related behaviors and falls in the elderly, and to provide clues for the prevention and intervention of injuries caused by falls in the elderly.

**Methods:**

Based on the basic demographic characteristics data, number of chronic diseases, health-related behaviors, and physical and mental health data of 5867 elderly people aged 60 and above in the 2018 CHARLS data, this paper used ordered logit regression to analyze the correlation between chronic diseases and their health-related behaviors and falls of Chinese elderly. On this basis, it also distinguishes whether there is care or not, explores whether the related factors of falls of elderly people will be different, and tests the intermediary effect of health status to further explore its mechanism.

**Results:**

The number of chronic diseases and health-related behaviors in the four dimensions of sleeptime, drinking, smoking, and activity are significantly correlated with falls in the elderly. Among them, health status plays a significant mediating role in the relationship of the number of chronic diseases and sleeptime and activity on the falls of the elderly. In addition, compared with the elderly without care, the risk of falls in the elderly in care is only related to the number of chronic diseases and sleeptime, while the elderly without care is related to the number of chronic diseases and multiple factors such as smoking, drinking and activity.

**Conclusion:**

Falls are significantly associated with chronic disease and health-related behaviors, while risk or protective factors for falls vary according to whether older adults are cared for. Therefore, targeted interventions can be made for the factors that affect the fall of the elderly according to different situations.

## Introduction

A fall is understood as “an event in which a person accidentally lies on the ground or at another lower level unaware of the loss of consciousness” [1]. It is one of the main causes of accidental injury, morbidity and even death of the elderly [2, 3]. In many countries, elderly falls have become a major public health problem [4], and have imposed huge financial and nursing burden [5]. After 60 years of age, the incidence and prevalence of falls and the severity of complications after falls gradually increase [6]. Accidental falls are the main cause of injuries or hospitalizations in Canadian elderly [7]. In China, about 50 million elderly people have at least one fall every year. 36–44% of patients go to the emergency room after a fall, followed by adverse events, including repeated falls, emergency room visits or death within 1 year [8]. In addition, China has a serious aging problem, and the prevalence of chronic diseases brought about by changes in population age structure tends to aggravate the inherent loss of structural and functional aging, which may lead to an increased risk of falls [9, 10]. Falling can aggravate and affect the quality of life of the elderly [11], it may seriously cause disability, loss of independence, fear of falling, social isolation and even death [12]. Therefore, it is necessary to explore the risk and protective factors of falling and conduct appropriate intervention.

Chronic diseases and falls are important health problems of the elderly, because they will reduce the quality of life [13]. Severe falls can lead to functional decline, loss of independence, and even death [14]. The main risk factors for falls interact in a complex way, including basic demographic characteristics: gender, age, residence, etc., and chronic disease is also a risk factor for falls [15]. For example, arthritis, diabetes, visual impairment, especially hypertension and chronic obstructive pulmonary disease, will lead to an increase in the number of falls in the elderly [16]. Canadian scholars have studied the rate and risk factors of falls in people with neurological diseases (such as dementia and Parkinson’s disease) [17, 18]. Chronic renal failure also increases the risk of falls [19]. Health-related behaviors are also risk or protective factors of falling, such as smoking, drinking, exercise [20], sleep [21]. A number of studies have explored the association between health status and falls in older adults. Different health status of the elderly has different risks of falls [22]. This has significant physical and psychological links to the elderly, as well as has a negative effect on their families and the wider community [23]. Elderly people with limited activities of daily living tend to be afraid of falls, physical activity is limited, physical function is weakened, and ultimately increase the risk of falls [24]. Mental health has also been identified as a major risk factor affecting falls. Relevant studies have shown that depression in the elderly will reduce cognitive function, affect physical function, and increase the risk of falls [25], that is, risk factors will affect falls in the elderly by affecting their physical health status [26].

In addition, studies have shown that care is also an important factor affecting elderly falls [27], but it is not clear whether the risk factors of accidental fall are different with or without care. Therefore, on the basis of exploring the risk factors affecting the fall of the elderly in different degrees, this paper also explores whether the risk factors of fall are different for the elderly with or without care.

Most of the above studies only explored the relationship between these risk factors and falls in the elderly, and did not in-depth study the internal mechanism of falls in the elderly. In order to solve the above problems, this study using the China Health and Retirement Longitudinal Study data to explore the correlation between different numbers of chronic diseases and their health-related behaviors on falls, and uses health status as the intermediary variable to conduct path analysis to further explore its inner mechanism, in-depth analysis of whether it is the health inequality caused by the relevant risk factors, and then the inequality of the elderly falling. At the same time, this paper also explores whether risk or protective factors for falls differ with or without care, with a view to opening up more windows of opportunity to intervene in the likelihood of related injuries [14].

## Method

### Data source

The data comes from China Health and Retirement Longitudinal Study (CHARLS) [28]. This project is a high-quality, representative household sample survey of households and individuals aged 45 and above in China conducted by the National Institute of Development of Peking University. The subjects covered 28 provinces in China, and the main purpose of this survey is to collect demographic information and basic information on health status of middle-aged and elderly people. A total of 19,816 pieces of data were collected from CHARLS data in 2018. After removing a large number of missing values, 5867 respondents were analyzed in this paper, including 2671 male and 3196 female respondents. In this study, the data from the CHARLS national survey were strictly sampled to avoid the problem of biased results due to the regional bias of the respondents as much as possible.

### Variable description

#### The dependent variable

The dependent variable is the self-reported fall problem of the elderly. In CHARLS’s 2018 survey, elderly fall questions included “Have you ever fallen?”. The answer options are binary choices, yes or no; And“ How many falls and injuries were serious enough to require medical attention? “. In this paper, the two problems are integrated, and the dependent variable “fall” is set as three categorical variables: “no fall, common fall and medical treatment for fall”.

#### The independent variables

The independent variables in this study are the number of chronic diseases and their health-related behaviors in the elderly. This paper synthesizes the questions and answers of 14 chronic diseases in the CHARLS questionnaire, and counts the number of people over 60 years old suffering from chronic diseases as one of the independent variables in this paper. In the existing papers, the definition of health-related behaviors has different emphasis [29, 30]. In general, smoking, drinking, activity and sleeptime are selected as health-related behaviors in this paper to explore the fall inequality of the elderly.The variable “activity” is measured by asking the respondents cyclically“whether they participate in vigorous-intensity activity, moderate activity and mild activities such as walking” through questionnaires. When the respondent participates in various intensity activities, the higher intensity activities is used as the activity situation, the three activities intensities are set to “vigorousactivity”, “moderateactivity” and “mildactivity”, which are assigned as 1, 2 and 3 respectively. Since we can’t know whether the reason for the respondents’ inactivity is that they are unable to exercise due to health problems or are not used to exercise, in order to avoid disputes over the reasons for not exercising, which may lead to erroneous estimation results, we excluded respondents who did not exercise. See Table 1 for other variable assignments.

**Table 1:** Variable description

Variable	Description of variable setting	Mean	Std. Dev.
Fall	No fall = 0, ordinary fall = 1, medical treatment for fall = 2	.3374808	.654105
Chronic Disease	The number of chronic diseases	.8448952	1.104562
Smoking	Yes = 1, quit = 2, no = 3	2.311062	.8677768
Drinking	Yes = 1, no = 2	1.695585	.4601982
Sleeptime	The amount of time you sleep each day	6.008928	2.065814
Activity	Vigorous-intensity activity = 1, Moderate activity =2, Mild activity = 3	2.078064	.8131051
Depression	The higher the number, the higher the depression level (range 0–30)	9.58991	6.76546
Pain	No = 0, yes = 1	.7030851	.4569377
ADL	ADL ability = 0, ADL disability = 1	.2657235	.4417554
Gender	Male = 1, female = 2	1.544742	.4980366
Age	60 ~ 69 = 1, 70 ~ 79 = 2, 80 ~ ~ = 3	1.506903	.656535
Education	Junior high school or below =1; Senior high school or above =2	1.100051	.3000937
Residence	Urban = 1, rural =2	1.721152	.4484707
Economic status	Continuous variable, take the log of economy	7.540106	3.073686
Marital status	Married = 1, single = 2	1.187489	.3903371
Care	With care =1, without care = 2	1.755071	.4300819

#### Mediating variables and control variables

With the change of disease spectrum and medical model, the traditional indicators for evaluating the relationship between health and disease, such as morbidity, cure rate and mortality rate, have been unable to comprehensively and deeply reflect the health status of patients with chronic diseases, and it is necessary to evaluate the health status of people from multiple perspectives such as physical and mental [31]. Considering the comprehensiveness of measuring health status and the research ability of setting variables in the questionnaire, this paper selects activities of daily living (ADL), pain status and depression status to comprehensively reflect the physical and mental health status of the elderly.

Activities of daily living (ADL) is measured according to Activity of Daily Living Scale developed by Lawton and Brody in the United States, which included six aspects: eating, dressing, getting up, going to the toilet, bathing and doing housework. The Cronbach’s alpha coefficient is 0.703, indicating good reliability and consistency. In order to achieve multivariate analysis as much as possible, this paper follows the previous research [32], using dichotomous variables, and setting the respondents who have no difficulties in the above six aspects as “ADL ability” with a value of 0, while the rest are set as “ADL disability” with a value of 1. The pain status mainly investigates whether the interviewee often feels bad because of pain. The choices are “Yes” and “No”, and they are assigned 1 and 0 respectively. Mental health is measured by the Depression Scale for the Elderly, which used the 10-question CES scale (center for epidemiologic studies-depression scale, CES-D) with a Cronbach alpha of 0.802. Contains ten questions with the following options: “rarely = 1”, “not too much = 2”, “sometimes = 3”, and “most = 4”. In this paper, an additive method was used to construct the depression index of the interviewees. The higher the value, the more serious the depression status is.

The control variables used the most basic demographic characteristics variables, including gender, age, education, residence and marital status. Other studies have shown that economic status and care can also affect the fall of the elderly [33, 34]. Therefore, in addition to the basic demographic statistical characteristic variables, this study also included economic status and care into the model to control their effects.

### The research methods

First of all, the variance analysis or chi-square test is used to make descriptive statistics on the variables to intuitively reflect the difference of falls among Chinese elderly people at different degrees. Next, mediating variables are used to analyze whether and how health-related behaviors in patients with chronic diseases affect falls in older Chinese. For the mediation analysis of dependent variables as category variables, this paper integrated the methods of scholars such as Wen ZhongLin [35], MacKinnon and Cox [36], and adopted a three-step method for testing. In the first step, Logit regression of dependent variable Y to independent variables X and M was performed. The second step is to do the regression with the dependent variable M and the independent variable X; Third, the significance of the mediating effect is tested by the Bias-corrected Bootstrap by the Stata software. As a repeat sampling estimation technique, this method can obtain more accurate interval estimates when monitoring indirect effects [37]. If the 95% confidence interval obtained by this method does not contain 0, it is proved that the mediation effect is significant; otherwise, the mediation effect is not significant.

Since the dependent variable in this paper is a classification variable, we use the ordered Logit regression model for data analysis. At the same time, the heterogeneity of care is analyzed on the basis of the ordered Logit regression model to further explore whether the risk factors of falling in the elderly are different with or without care.

Then, the path analysis method is used to further investigate the influence of the number of chronic diseases and health-related behaviors on the fall of the elderly, and more intuitively show how each factor affects the fall of the elderly to different degrees.

Finally, the Probit model is used for robustness test to reconfirm the correlation between the number of chronic diseases and health-related behaviors on falls in the elderly, so as to ensure the reliability of the study results.

**Fig. 1:**
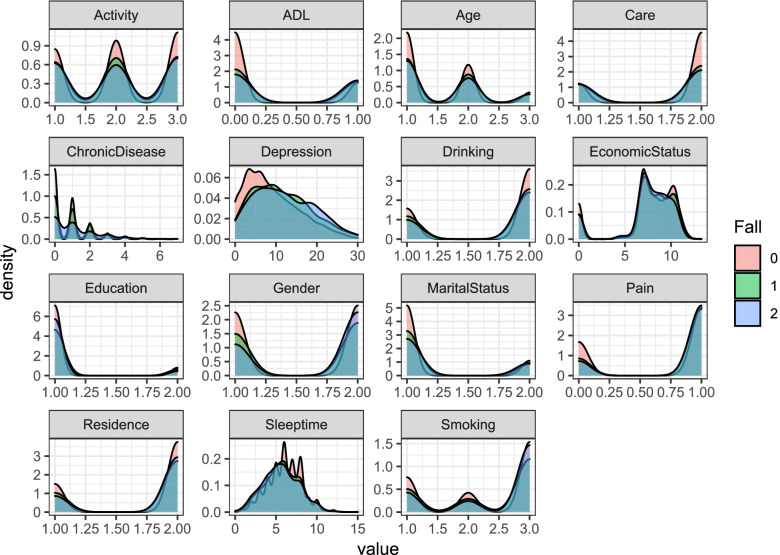
Density curves of each variable under the probability of falling to different degrees. Note: ^1^ 0, 1 and 2 in the figure are used to indicate different categories of falls (No fall = 0, ordinary fall = 1, medical treatment for fall = 2). ^2^ADL (Activity of Daily Living) is used to describe the daily living ability of the elderly

**Fig. 2:**
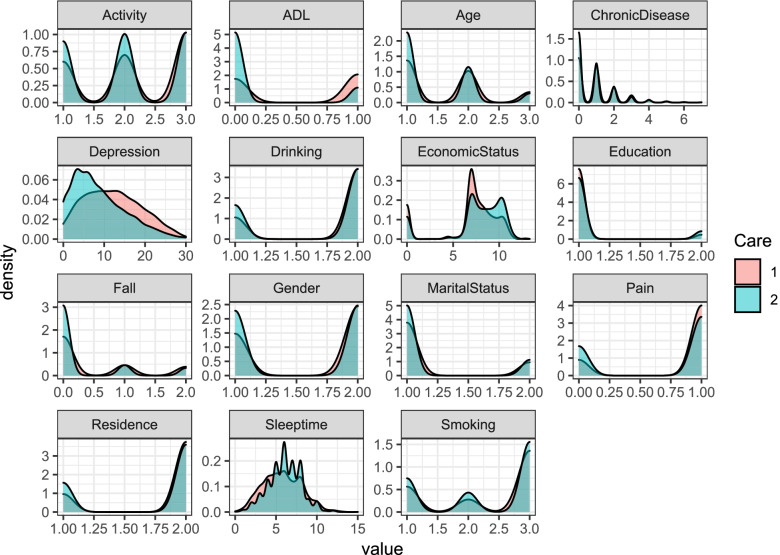
Density curve of each variable with or without care. Note: ^1^1 and 2 in the figure are used to indicate the different categories of care (with care = 1 without care = 2). ^2^ADL (Activity of Daily Living) is used to describe the daily living ability of the elderly

In this paper, variance analysis, chi-square test, regression analysis, and mediation effect test are all performed in Stata; the density curves (Fig. 1, Fig. 2) are generated by the ggplot2 package in the R software; and the internal consistency test is completed in SPSS.

## Results

### Respondents characteristics distribution

#### Descriptive statistics of falls in different severity in the elderly

Table 2 gives the basic information of all respondents in this paper. Among the respondents selected in this article, 76.46% of elderly people who have not fallen, 782 respondents of ordinary elderly people who have fallen, accounting for 13.33, and 10.21% (599) of elderly people who need to seek medical treatment after a fall, which is similar to the proportion of the elderly who sought medical treatment after falling in previous studies [8]. The proportion of the young (60–70 years old) is relatively high, the elderly (over 80 years old) is relatively low, the elderly female is slightly higher than the elderly male, the education level is mostly concentrated in junior high school or below, the education level is generally low. The distribution of these basic characteristics is basically consistent with the survey data of the National Bureau of Statistics of China. In other words, the respondents selected in this paper have good representativeness. Descriptive statistical results showed that the number of chronic diseases, smoking, sleeptime, ADL, pain, depression had statistical significance among the elderly with different severity of falls (all *P* < 0.5).

**Table 2:** Descriptive statistics of falls in different severity in the elderly

Variable		All	No fall	Ordinary fall	Medical treatment for fall	Chi-square	*P* Value
**The independent variable**							
ChronicDisease^a^	–	5867	4486 (76.46)	782 (13.33)	599 (10.21)	43.2881	0.000
Smoking	Yes	1580 (26.93)	1258 (28.04)	202 (25.83)	120 (20.03)	33.0903	0.000
	Quit	882 (15.03)	698 (15.56)	116 (14.83)	68 (11.35)		
	No	3405 (58.04)	2530 (56.40)	464 (59.34)	411 (68.61)		
Drinking	Yes	1786 (30.44)	1363 (30.38)	257 (32.86)	166 (27.71)	4.2815	0.118
	No	4081 (69.56)	3123 (69.62)	525 (67.14)	433 (72.29)		
Sleeptime^a^	–	5867	4486 (76.46)	782 (13.33)	599 (10.21)	10.8246	0.004
Activity	Vigorous- intensity	1728 (29.45)	1292 (28.80)	242 (30.95)	194 (32.39)	5.6334	0.228
	Moderate	1953 (33.29)	1500 (33.44)	267 (34.14)	186 (31.05)		
	Mild	2186 (37.26)	1694 (37.76)	273 (34.91)	219 (36.56)		
**Mediating variables**							
ADL	ability	4308 (73.43)	3493 (77.86)	480 (61.38)	335 (55.93)	197.4529	0.000
	disability	1559 (26.57)	993 (22.14)	302 (38.62)	264 (44.07)		
Pain	No	1742 (29.69)	1479 (32.97)	160 (20.46)	103 (17.20)	99.8146	0.000
	Yes	4125 (70.31)	3007 (67.03)	622 (79.54)	496 (82.80)		
Depression^a^	–	5867	4486 (76.46)	782 (13.33)	599 (10.21)	13.8202	0.001
**Control variables**							
Age	60–70	3424 (58.36)	2663 (59.36)	426 (54.48)	335 (55.93)	11.1386	0.025
	70 ~ 80	1912 (32.59)	1440 (32.10)	275 (35.17)	197 (32.89)		
	80~	531 (9.05)	383 (8.54)	81 (10.36)	67 (11.19)		
Gender	male	2671 (45.53)	2127 (47.41)	346 (44.25)	198 (33.06)	44.5303	0.000
	female	3196 (54.47)	2359 (52.59)	436 (55.75)	401 (66.94)		
Residence	urban	1636 (27.88)	1286 (28.67)	214 (27.37)	136 (22.70)	9.4631	0.009
	rural	4231 (72.12)	3200 (71.33)	568 (72.63)	463 (77.30)		
Education	junior high school or below	5280 (89.99)	4033 (89.90)	691 (88.36)	556 (92.82)	7.6701	0.022
	senior high school or above	587 (10.01)	453 (10.10)	91 (11.64)	43 (7.18)		
Marital status	married	4767 (81.25)	3702 (82.52)	620 (79.28)	445 (74.29)	25.8044	0.000
	single	1100 (18.75)	784 (17.48)	162 (20.72)	154 (25.71)		
Care	with care	1437 (24.49)	961 (21.42)	258 (32.99)	218 (36.39)	99.2929	0.000
	without care	4430 (75.51)	3525 (78.58)	524 (67.01)	381 (63.61)		
Economic status^a^	–	5867	4486 (76.46)	782 (13.33)	599 (10.21)	4.1414	0.042

Marked ^a^ is continuous variable, which is analyzed by variance, and the rest are classified variable, which is tested by chi-square test

#### Characteristic distribution of each variable under different degree of fall probability

Figure 1 shows the density distribution of each variable in different groups of elderly people who did not fall,ordinary fall, and medical treatment for fall, which can more intuitively reflect the distribution difference of each variable in elderly groups with different degrees of falls. As can be seen from Fig. 1, among the independent variables, the number of chronic diseases, sleeptime and activity have significant differences in the fall degree of the elderly, while smoking and drinking have certain differences in the fall degree of the elderly. Among the mediating variables, ADL, pain and depression are associated with falls in the elderly. There are big differences in degree; among the control variables, age, gender, marital status, education, and care of the elderly have great differences in different degrees of falls, and economic status has certain differences in the degree of elderly falls.

### The association between falls and the number of chronic diseases, health-related behaviors, and health status

#### The correlation between the number of chronic diseases, health-related behaviors and different degrees of falls in the elderly

Table 3 presents the correlation between the number of chronic diseases and health-related behaviors estimated by the ordered Logit regression model on falls of the elderly to different degrees. Model 1, Model 3 and Model 5 are regression estimates of the number of chronic diseases, health-related behaviors and control variables on falls of the elderly to different degrees. Model 2, Model 4 and Model 6 are full models with mediating variables. Model 1 showed that the number of chronic diseases and smoking have a significant positive correlation (*p* < 0.01) on falls of the elderly in different degrees. That is, the more the number of chronic diseases in the elderly, the higher the probability of falling. Specifically, the probability of falling or going to the doctor increased by 0.167 for each unit of the number of chronic diseases in the elderly. The respondents who don’t smoke have an increased severity of falls compared with those who smoke, with an increased probability of 0.104 (*p* < 0.05). However, sleeptime, drinking and activity have a significant negative correlation with different degrees of falls in the elderly (*p* < 0.01). For each additional unit of sleep duration, the probability of falling or seeking medical attention for the elderly will decrease by 0.0725; for the elderly who drink alcohol, the probability of falling or seeking medical attention will decrease by 0.224; for each level of decrease in the activity intensity of the elderly, the risk of falling or seeking medical attention will decrease by 0.104. After adding the intermediary variable (health status) in Model 2, the correlations between each core independent variable and falls are similar to Model 1.

**Table 3:** Logit regression estimation of the number of chronic diseases, health behaviors and falls in the elderly

	All		Care (yes)		Care (no)	
	m1	m2	m3	m4	m5	m6
Variables	Fall	Fall	Fall	Fall	Fall	Fall
**ChronicDisease**	0.167*** (0.0265)	0.113*** (0.0272)	0.163*** (0.0461)	0.129*** (0.0470)	0.165*** (0.0326)	0.102*** (0.0336)
**Sleeptime**	−0.0725*** (0.0150)	− 0.0324** (0.0154)	− 0.0784*** (0.0245)	− 0.0533** (0.0252)	− 0.0691*** (0.0191)	−0.0203 (0.0196)
**Drinking**	−0.224*** (0.0741)	−0.229*** (0.0750)	− 0.126 (0.138)	−0.0914 (0.140)	− 0.278*** (0.0883)	−0.301*** (0.0895)
**Smoking**	0.104** (0.0489)	0.110** (0.0492)	0.0311 (0.0852)	0.0268 (0.0858)	0.141** (0.0598)	0.155** (0.0603)
**Activity**	−0.104*** (0.0400)	− 0.104** (0.0406)	− 0.0918 (0.0714)	− 0.123* (0.0729)	− 0.107** (0.0485)	−0.0936* (0.0492)
**Age**	0.109** (0.0487)	0.119** (0.0494)	−0.0241 (0.0850)	0.0239 (0.0868)	0.167*** (0.0594)	0.154** (0.0605)
**Gender**	0.234*** (0.0886)	0.156* (0.0897)	0.0935 (0.160)	0.0752 (0.162)	0.303*** (0.107)	0.198* (0.108)
**Residence**	0.187** (0.0790)	0.0710 (0.0804)	−0.0150 (0.148)	−0.0734 (0.150)	0.268*** (0.0938)	0.125 (0.0958)
**Education**	0.115 (0.111)	0.177 (0.112)	0.161 (0.240)	0.205 (0.243)	0.109 (0.126)	0.174 (0.128)
**Marital status**	0.280*** (0.0794)	0.217*** (0.0809)	0.463*** (0.142)	0.433*** (0.143)	0.204** (0.0965)	0.116 (0.0988)
**Economic status**	0.0103 (0.0109)	0.0186* (0.0112)	0.0120 (0.0189)	0.0200 (0.0191)	0.00902 (0.0134)	0.0170 (0.0138)
**Care**	−0.590*** (0.0692)	− 0.293*** (0.0742)				
**Depression**		0.0284*** (0.00497)		0.0218** (0.00873)		0.0320*** (0.00604)
**Pain**		0.414*** (0.0800)		0.165 (0.161)		0.494*** (0.0927)
**ADL**		0.559*** (0.0724)		0.492*** (0.119)		0.590*** (0.0914)
**/cut1**	0.927*** (0.351)	2.153*** (0.369)	0.974 (0.614)	1.763*** (0.648)	2.344*** (0.387)	2.986*** (0.400)
**/cut2**	1.955*** (0.353)	3.204*** (0.371)	2.018*** (0.617)	2.825*** (0.651)	3.366*** (0.389)	4.035*** (0.403)
**Observations**	5867	5867	1437	1437	4430	4430

z-statistics in parentheses; *** *p* < 0.01, ** *p* < 0.05, * *p* < 0.1

Model 3 shows that the increase in the risk of falling for the elderly in care is only related to the increase in the number of chronic diseases (*r* = 0.163, *p* < 0.01) and the reduction in sleeptime (*r* = − 0.0784, *p* < 0.01). After health status is added into model 4, the results show that mild activity will also reduce the probability of falling in the elderly (*r* = − 0.123, *p* < 0.1), and whether it is painful the relationship with the elderly’s fall risk disappeared.

The risk factors for falling are different for those who were not cared for compared with those who were. Results of Model 5 shows that the risk of falling is correlated with the number of chronic diseases, sleeptime, drinking, smoking and physical activity in the elderly without care, and the direction of influence is similar to Model 1. When health status is included, regression model six showed that only sleeptime is not significantly associated with fall risk in older adults.

Figure 2 shows the distribution difference between the characteristics of each variable and whether the elderly are cared for. In terms of sleeptime, activity, depression, pain, ADL, economic status and other characteristics, the distribution of whether they are cared for is very obvious, while in terms of the number of chronic diseases, education, smoking and other characteristics, the distribution of care of the elderly is only slightly different.

#### The correlation between the number of chronic diseases and health-related behaviors and the health status of the elderly

The correlation between the number of chronic diseases and health-related behaviors on falls of the elderly to different degrees have been confirmed above, but there is no association between the number of chronic diseases and health-related behaviors on the health status of the elderly. In order to further confirm the association between the number of chronic diseases, health-related behavior,health status and falls, the Logit regression model of the number of chronic diseases and health-related behavior variables and control variables on the three indicators of the elderly’s health status is constructed below (Table 4). Considering the simplicity of the table and the convenience of analysis, Table 4 only provides the estimated results of the number of chronic diseases and the core independent variables of health-related behaviors for the health status of the elderly. On this basis, we also consider whether the heterogeneous factor of care would be associated with health status.

**Table 4:** Logit regression estimation of the number of chronic diseases, health behaviors and health status in the elderly

		(1)	(2)	(3)
	Variables	Depression	Pain	ADL
All	ChronicDisease	0.688*** (0.0742)	0.329*** (0.0320)	0.186*** (0.0280)
	Sleeptime	− 0.721*** (0.0398)	−0.130*** (0.0151)	− 0.107*** (0.0154)
	Drinking	0.528*** (0.194)	−0.00857 (0.0698)	− 0.0346 (0.0778)
	Smoking	−0.172 (0.126)	− 0.0433 (0.0455)	0.0221 (0.0503)
	Activity	−0.107 (0.104)	−0.208*** (0.0388)	0.132*** (0.0412)
Care (yes)	ChronicDisease	0.571*** (0.147)	0.162*** (0.0474)	0.139*** (0.0399)
	Sleeptime	−0.678*** (0.0755)	− 0.0647*** (0.0235)	− 0.176*** (0.0207)
	Drinking	0.273 (0.438)	−0.191 (0.136)	0.0779 (0.114)
	Smoking	0.131 (0.268)	−0.0217 (0.0832)	0.0253 (0.0693)
	Activity	0.282 (0.223)	0.250*** (0.0694)	0.0615 (0.0586)
Care (no)	ChronicDisease	0.731*** (0.0860)	0.316*** (0.0353)	0.193*** (0.0351)
	Sleeptime	−0.740*** (0.0469)	−0.135*** (0.0173)	−0.139*** (0.0204)
	Drinking	0.580*** (0.215)	0.0253 (0.0762)	0.00559 (0.0970)
	Smoking	−0.287** (0.142)	−0.0159 (0.0501)	0.0431 (0.0641)
	Activity	−0.223* (0.118)	− 0.163*** (0.0426)	0.0774 (0.0517)

1 z-statistics in parentheses *** *p* < 0.01, ** *p* < 0.05, * *p* < 0.1

2 Depression is a continuous variable, and OLS regression model is used, while other variables are classified variables, and Logit model is used

3 Control variables are not placed in the table for simplicity

In the regression model that included all respondents, the number of chronic diseases have a significant positive correlation with the health status of the elderly (*P* < 0.01). For each unit increase in the number of chronic diseases in the elderly, their pain, ADL status become worse, and depression is deepened. The probability of sleep increases by 0.329, 0.186 and 0.688, respectively; sleeptime is significantly negatively correlated with health status (*P* < 0.01), and each unit of sleeptime increases the probability of pain, worse ADL status, and deepening depression. 0.130, 0.107 and 0.721. Compared with the elderly who drink alcohol, the probability of deepening depression of the elderly who do not drink will increase by 0.528 (*p* < 0.01). Compared with the elderly who do vigorous-intensity activity, doing mild activity such as walking will reduce the pain of the elderly. At the same time, the elderly who do vigorous-intensity activity have stronger ADL ability.

In the respondents with care and without care, the number of chronic diseases and sleeptime show significant correlations with the three health status indicators of the elderly, which is similar to the regression model that included all respondents. The difference is that smoking, drinking and activity are significantly associated with depression among the elderly without care compared with those with care. Among the respondents without care, the non-drinking respondents have a 0.580 (*p* < 0.01) increased probability of depression compared with the alcohol-drinking respondents. The respondents who don’t smoke and do mild activity are 0.287 and 0.223 less likely to be depressed than those who smoke and do vigorous-intensity activity.

### The mediating effect of health status on the number of chronic diseases, health-related behaviors and falls in the elderly

It can be seen from the foregoing that the number of chronic diseases and health-related behaviors in the elderly have varying degrees of influence on the fall risk of the elderly. In order to further explore the influence mechanism of various variables on the fall risk of the elderly, path analysis is adopted to explore the influence of the number of chronic diseases and health-related behaviors on the fall risk of the elderly.

The Bootstrap method with bias correction is used to test the significance of the mediating effect, and the results are shown in Table 5. The 95%Bootstrap confidence intervals of the number of chronic diseases affecting the fall of the elderly at different degrees through depression, pain and ADL are [0.0062, 0.0116], [0.0053, 0.0099] and [0.0047, 0.0099] respectively, all excluding 0, indicating a significant mediating effect. That is, an increase in the number of chronic diseases in the elderly could further increase the risk of falls by affecting their health. The 95%Bootstrap confidence intervals of sleep duration affecting the fall of the elderly at different degrees through depression, pain and ADL are [− 0.0108, − 0.0061], [− 0.0048, − 0.0025] and [− 0.0055, − 0.0027], respectively, excluding 0, showing a mediating effect. That is, sleeptime further affect falls in older people by affecting their health. The 95% Bootstrap confidence intervals that activity affects different degrees of falls in the elderly through pain and ADL are [− 0.0083, − 0.0034], and [0.0013, 0.0080], respectively, excluding 0, and there is a mediating effect. That is to say, there is a certain correlation between the activity intensity of the elderly and pain, ADL and falls of the elderly. The health status of the elderly do not play a significant mediating role between smoking and falls.

**Table 5:** Test results of mediating effect

Mediation model	Mediating effect estimation		95%Bootstrap Conf. interval	
	Coef.	Std.Err	Lower limits	Upper limits
**CD-Depression-Fall**	0.0089***	0.0014	0.0062	0.0116
**CD-Pain-Fall**	0.0076***	0.0012	0.0053	0.0099
**CD-ADL-Fall**	0.0073***	0.0013	0.0047	0.0099
**Sleeptime-Depression-Fall**	− 0.0085***	0.0012	−0.0108	− 0.0061
**Sleeptime -Pain-Fall**	−0.0036***	0.0006	−0.0048	− 0.0025
**Sleeptime-ADL-Fall**	− 0.0041***	0.0007	−0.0055	− 0.0027
**Drinking-Depression-Fall**	0.0056**	0.0024	0.0010	0.0103
**Drinking -Pain-Fall**	−0.0012	0.0021	−0.0053	0.0030
**Drinking -ADL-Fall**	−0.0004	0.0027	− 0.0056	0.0048
**Smoking-Depression-Fall**	−0.0018	0.0016	−0.0049	0.0013
**Smoking -Pain-Fall**	−0.0012	0.0014	−0.0039	0.0015
**Smoking -ADL-Fall**	0.0007	0.0018	−0.0028	0.0042
**Activity-Depression-Fall**	−0.0018	0.0014	−0.0045	0.0009
**Activity -Pain-Fall**	−0.0058***	0.0012	−0.0083	− 0.0034
**Activity -ADL-Fall**	0.0046***	0.0017	0.0013	0.0080

1 *** *p* < 0.01, ** *p* < 0.05, *p* < 0.1; Bootstrap sampling number = 1000

2* CD* Chronic Disease, *ADL*  Activity of Daily Living

### Robustness test

Next, Probit models is used to test the robustness of the model. Model 3 and Model 4 are Probit regression before and after adding mediation variables, and the significance of the core independent variables and mediation variables is similar to the results of logit regression. The results of Probit regression is basically consistent with the analysis results of Logit regression model mentioned above, that is, the results of this study have strong robustness, the specific results are shown in Table 6.

**Table 6:** Analysis results of Probit regression models

	Logit		Probit	
	m1	m2	m4	m5
Variables	Fall	Fall	Fall	Fall
ChronicDisease	0.167*** (0.0265)	0.113*** (0.0272)	0.0951*** (0.0161)	0.0615*** (0.0166)
Sleeptime	− 0.0725*** (0.0150)	− 0.0324** (0.0154)	− 0.0418*** (0.00888)	−0.0182** (0.00922)
Drinking	−0.224*** (0.0741)	−0.229*** (0.0750)	− 0.135*** (0.0438)	− 0.140*** (0.0443)
Smoking	0.104** (0.0489)	0.110** (0.0492)	0.0582** (0.0287)	0.0637** (0.0290)
Activity	−0.104*** (0.0400)	−0.104** (0.0406)	− 0.0637*** (0.0236)	− 0.0629*** (0.0240)
Age	0.109** (0.0487)	0.119** (0.0494)	0.0701** (0.0289)	0.0768*** (0.0293)
Gender	0.234*** (0.0886)	0.156* (0.0897)	0.125** (0.0524)	0.0732 (0.0532)
Residence	0.187** (0.0790)	0.0710 (0.0804)	0.0990** (0.0461)	0.0237 (0.0470)
Education	0.115 (0.111)	0.177 (0.112)	0.0844 (0.0652)	0.121* (0.0660)
Marital status	0.280*** (0.0794)	0.217*** (0.0809)	0.151*** (0.0479)	0.114** (0.0487)
Care	−0.590*** (0.0692)	−0.293*** (0.0742)	−0.355*** (0.0419)	−0.182*** (0.0449)
Economic status	0.0103 (0.0109)	0.0186* (0.0112)	0.00537 (0.00636)	0.0103 (0.00648)
Depression		0.0284*** (0.00497)		0.0173*** (0.00300)
Pain		0.414*** (0.0800)		0.235*** (0.0453)
ADL		0.559*** (0.0724)		0.332*** (0.0441)
	0.927*** (0.351)	2.153*** (0.369)		
/cut2	1.955*** (0.353)	3.204*** (0.371)		
Constant			−0.507** (0.207)	−1.204*** (0.217)
Observations	5867	5867	5867	5867

z-statistics in parentheses;*** *p* < 0.01, ** *p* < 0.05, * *p* < 0.1

## Discussion

As the primary cause of non-accidental injuries among the elderly in China, falling may cause injuries of different severity. At present, there are few reports on the incidence of falls among the elderly with different severity in China. Based on the data of CHARLS 2018, this paper shows that the common fall rate and the hospitalization rate of the elderly in China are 13.33 and 10.21% respectively. In this study, ordered Logit regression is used to explore the influencing factors of falls of different severity in the elderly, to identify the significant effects of chronic diseases、health-related behaviors and health status on falls in the elderly, and to compare whether the risk of falls in the elderly differs with care through differential analysis.

Studies have shown that there is a significant correlation between the number of chronic diseases in the elderly and the risk of falls in the elderly. The greater the number of chronic diseases in the elderly, the greater the risk of falls [16]. Meanwhile, through mediating effect test, it is found that health status plays a significant mediating role between the number of elderly patients and falls. The more the number of chronic diseases the elderly suffered from, the worse their health status is and the higher the risk of falls, which is basically consistent with the research results of Moylan and Binder [38]. Personal health-related behaviors are associated with the risk of falling or injury when falling. An unhealthy lifestyle is likely to cause serious consequences of falling [39], but the path of its association on falling is not clear. We find that sleeptime, drinking, smoking, and activity have significant correlations with fall risk in the elderly, while differences in sleeptime and activity cause health differences in the elderly, and thus has a strong indirect effect on falls in the elderly. Poor sleep quality is associated with an increased risk of falls and hospitalization among older Chinese. Sleep disorders are potentially remediable risk factors and need more attention in epidemiological studies. Identifying sleep disorders may help identify high-risk Chinese elderly people who may benefit from education to prevent falls [21]. Activity is a protective factor for falls. Keeping physical activity can not only improve physical condition [40], but also have broader benefits, such as expansion of social functions [41], which can effectively prevent falls. This article finds that compared with intensity activity, the risk of mild activity of the elderly fell significantly reduce. The possible reason is that these respondents are weak, medically unstable, restricted in movement. Mild exercise does not show significant health improvement [42] and is more likely to cause falls, while those who do severe exercise maintain better physical functions and are less likely to fall. Current studies, using population-based data, have identified daily alcohol intake as a risk factor for non-fatal fall injuries in general and in certain subtypes, high-risk drinkers are more likely to suffer non-fatal fall injuries than lifelong teetotalers [43], and excessive alcohol consumption can even lead to hospitalization in the elderly [44]. And the reason that alcohol consumption directly affects falls in older adults, rather than through health and falling as expected, is that most unhealthy older adults drink less or no alcohol, while healthy older adults drink moderately. “Healthy drinkers” [43]. Low-risk drinking and its healthy drinking patterns may have minimal correlation with health [45]. Older people who don’t smoke have more severe falls than older people who smoke. This may be because non-smokers may be exposed to smoking-related environmental stimuli, especially secondhand smoke exposure, even though they do not smoke at all. According to the survey of Chinese Center for Disease Control and Prevention, 76.3, 57.1 and 54.3% of respondents reported that they were exposed to second-hand smoke in restaurants, homes and workplaces [46]. Compared with the mainstream smoke inhaled by smokers, the chemical composition and concentration of tobacco smoke inhaled by second-hand smoke exposure are different. The content of some chemical components that are seriously harmful to human body in secondhand smoke is even higher than that in mainstream smoke. Among them, the content of carbon monoxide, nicotine, benzo pyrene and nitrosamine, which are strongly carcinogenic, are 5 times, 3 times, 4 times and 50 times of the content of mainstream smoke respectively. Exposure to secondhand smoke can cause serious damage to human health, and there is extensive evidence that secondhand smoke can cause a number of unexpected health hazards, including an increased risk of cardiovascular disease, cancer, and respiratory diseases in adults.

There is a significant difference in fall problems between the elderly who are cared for and those who are not. The elderly in care is at greater risk of falls, possibly because the elderly in need of care themselves have poorer health conditions, such as functional limitations, cognitive impairments, decreased strength and balance, chronic pain or comorbidities [34]. compared with the elderly without care, the increase in the risk of falling is only associated with the increase in the number of chronic diseases and the decrease in the sleeptime of the elderly in care, while the factors affecting the fall of the elderly without care are affected by the number of chronic diseases and various factors such as smoking, drinking and activity.

The weakness of this paper is that we used cross-sectional data and could not prove the causal relationship between the number of chronic disease,health-related behaviors, health status and falls of the elderly. Moreover, our respondents are influenced by healthy respondents, leading to greater participation of healthier individuals, especially in the group of smokers and drinkers, that is, smoking and drinking may be only one indicator of health. In fact, the correlation between alcohol and tobacco on health and falls is quite strong, and the analysis has been adjusted for many confounding factors, indicating that these biases cannot fully explain the observed results. At the same time, due to the limitation of respondents data, there are more possible factors that have not been considered. For example, whether the elderly use drugs and the different types of drugs, whether the use of health care products will have a association with the elderly fall; In addition, the difference of living environment (whether there are dangerous objects that may cause falls in the home), gait, balance and so on, whether and how the elderly fall will have an effect can be further explored.

## Conclusion

In this paper, we find that elderly patients with chronic diseases and their unhealthy behaviors increase the risk of falls. Morbidity is a risk factor for common falls and medical treatment in the elderly. Adequate sleep and moderate activity are protective factors for common falls and hospitalization of the elderly. In addition, for the elderly with or without care, the factors that affect the risk of falls are different. We have not yet realized this, but still treat the elderly group as a whole. In this regard, targeted interventions can be made for the factors that affect the fall of the elderly according to different situations. For the elderly in care, we should pay more attention to their sleep conditions. At the same time, strengthen chronic disease management and deepen health education. Comprehensive treatment, prevention and control of chronic diseases should be done to prevent the “blowout” of chronic diseases in the future. As for the elderly who are not under care, in addition to focusing on their sleep and chronic diseases, they should also intervene in health-related behaviors such as smoking, drinking, and exercise. Promote or encourage the elderly with weaker physique to exercise moderately and reduce the frequency of drinking and smoking.

## Data Availability

The datasets supporting the study are publicly available on the CHARLS website http://charls.pku.edu.cn/index/zh-cn.html

## Abbreviations

CHARLS: China Health and Retirement Longitudinal Study
ADL: Activity of Daily Living
